# Supplementary material for: Complex charge density waves in simple electronic systems of two-dimensional III2–VI3 materials
Source: Nat Commun. 2024 Nov 18;15:9983. doi: 10.1038/s41467-024-54205-1 (PMC11574180; doi:10.1038/s41467-024-54205-1)
Supplement: Supplementary file 1 — Supplementary Information [file 41467_2024_54205_MOESM1_ESM.pdf]

Supplementary Information for

"Complex charge density waves in simple electronic systems of two-dimensional III_2–VI_3 materials"

Yu-Ting Huang[1], Zhen-Ze Li[1,2], Nian-Ke Chen[1,*], Yeliang Wang[3], Hong-Bo Sun[1,2], Shengbai Zhang[4], and Xian-Bin Li[1,*]

AFFILIATIONS

[1]*State Key Laboratory of Integrated Optoelectronics, College of Electronic Science and Engineering, Jilin University, 130012 Changchun, China*

[2]*State Key Laboratory of Precision Measurement Technology and Instruments, Department of Precision Instrument, Tsinghua University, 100084 Beijing, China*

[3]*School of Integrated Circuits and Electronics, MIIT Key Laboratory for Low-Dimensional Quantum Structure and Devices, Beijing Institute of Technology, Beijing, 100081 China*

[4]*Department of Physics, Applied Physics, and Astronomy, Rensselaer Polytechnic Institute, Troy, New York 12180, USA*

*Corresponding authors: N.C. (chennianke@jlu.edu.cn), X.L. (lixianbin@jlu.edu.cn)

CONTENTS

Supplementary Note 1. Computational details.

Supplementary Note 2. Partial density of states of pristine high-symmetry (β_c) and $\sqrt{13}\times\sqrt{13}$ chiral Star-of-David (c-SoD) CDW phases of 2D In_2Se_3.

Supplementary Note 3. Molecular dynamics simulation of monolayer c-SoD β-In_2Se_3 at 200 K.

Supplementary Note 4. Eigen motion analysis of representative phonon modes with imaginary frequencies for 2D β_c-In_2Se_3.

Supplementary Note 5. Antichiral SoD CDW phase of 2D β-In_2Se_3.

Supplementary Note 6. The relaxed atomic structures of different CDW orders of 2D β-In_2Se_3.

Supplementary Note 7. Density functional theory band structures of 2D c-SoD In_2Se_3 using different pseudopotentials.

Supplementary Note 8. Charge density differences of the β_c and c-SoD CDW phases of 2D In_2Se_3.

Supplementary Note 9. PBE band structures of different CDW phases of 2D β-In_2Se_3.

Supplementary Note 10. Structures of c-SoD CDW phase in nine 2D III_2–VI_3 materials.

Supplementary Note 11. PBE and HSE06 band structures of β_c and c-SoD phases of 2D III_2–VI_3.

Supplementary Note 1. Computational details.

First-principles calculations

The Vienna ab initio simulation package (VASP) was used for density functional theory (DFT) calculations[1]. The projector augmented wave (PAW) pseudopotential was used and the Perdew-Burke-Ernzerhof (PBE) exchange-correlation functional was employed[2,3]. Since the usual PBE scheme is known to underestimate the bandgap, the Hyed-Scuseria-Ernzerhof (HSE06) hybrid functional was also used to calculate the band structure of β_c and c-SoD phases of monolayer III_2–VI_3 with the mixing parameters set to 25%[4]. The outmost *s* and *p* electrons for S, Se, Te, Al, and *s*, *p*, *d* electrons for Ga and In are considered as valence states in the PAW potentials. The energy cut-offs of the plane wave basis sets were set as follows: 364 eV for Al_2S_3, 312 eV for Al_2Se_3, 312 eV for Al_2Te_3, 367 eV for Ga_2S_3, 367 eV for Ga_2Se_3, 367 eV for Ga_2Te_3, 364 eV for In_2S_3, 311 eV for In_2Se_3, 311 eV for In_2Te_3. A vacuum layer with a thickness of 20 Å was constructed in the *z*-direction for all the cells in this study to minimize the interactions between periodic images introduced by periodic boundary conditions. Electronic minimization was set to a tolerance of 10^{-6} eV, and ionic relaxation used a force tolerance of 0.01 eV/Å on each ion.

STM simulations

The simulated STM patterns were obtained using the VASP[1] package and the VASPKIT[5] processing program. First, self-consistent calculations were performed using the VASP software package, and the wave function (WAVECAR) and charge density (CHGCAR) files were generated. Next, non-self-consistent calculations were performed by VASP to obtain the partial charge density file (PARCHG). Finally, the VASPKIT code was used to process the PARCHG file based on the Tersoff-Hamann approximation[6] to generate STM images.

Energy comparison of various phases of 2D In_2Se_3

When comparing the relative energies of different phases of 2D In_2Se_3, finer *k*-point meshes were used. For the geometry optimization, the tolerance of force on each ion

was 0.01 eV/Å and the energy cutoff was 311 eV, the *k*-point meshes were set as follows: 13×13×1 for β_c and FE phases, 7×13×1 for AFE_1 phase, 4×13×1 for AFE_2 phase, 3×13×1 for AFE_3 phase, 2×13×1 for AFE_4 phase, 7×9×1 for $2\times\sqrt{3}$ phase, 5×5×1 for $\sqrt{7}\times\sqrt{7}$ phase, 4×4×1 for c-SoD phase, respectively. For static calculations, the *k*-point meshes were: 17×17×1 for β_c and FE phases, 10×17×1 for AFE_1 phase, 5×17×1 for AFE_2 phase, 4×17×1 for AFE_3 phase, 2×17×1 for AFE_4 phase, 10×13×1 for $2\times\sqrt{3}$ phase, 7×7×1 for $\sqrt{7}\times\sqrt{7}$ phase, 6×6×1 for c-SoD phase, respectively.

Molecular dynamics

The *ab initio* molecular dynamics (MD) simulations of monolayer c-SoD In_2Se_3 were performed using the VASP package. The NVT ensemble with Nosé thermostat was used[7]. A supercell containing 260 atoms was constructed for MD simulations and the k-point mesh is 1×1×1. The time step is set to 1 fs, and the simulation was conducted for 10000 steps at 200 K.

Phonon calculations

The phonon band structure of monolayer β_c-In_2Se_3 in our work was calculated using the finite displacement method. The computational parameters of phonon band structure are rigorously tested for convergence, and the specific parameters and results are shown in Supplementary Fig. 1 and Supplementary Table 1. As we can see from the convergence test, here with four sets of different calculation parameters, the phonon band dispersion exhibits negligible differences. Especially, the flat soft phonon modes along the Γ-M path, which induces the CDW orders, can be observed in all the cases. Finally, the force convergence tolerance for geometry optimization was 10^{-8} eV/Å, and the *k*-point mesh was 21×21×1 for the unit cell. The interatomic forces were computed using 6×6×1 supercell and 1×1×1 *k*-point mesh, with the tolerance for the convergence of total energy set to 10^{-8} eV. The force constant and the final phonon band structure were obtained using the Phonopy code[8].

Supplementary Table 1. The detailed parameters of the phonon band structure calculations for the convergence test in **Supplementary Figures 1(a-d)**.

Calculation	Force for geometry optimization	k-point mesh for geometry optimization (Unit cell)	k-point mesh for interatomic force (Supercell)
Supplementary Figure 1a	10^{-6} eV/Å	13×13×1	1×1×1
Supplementary Figure 1b	10^{-7} eV/Å	16×16×1	1×1×1
Supplementary Figure 1c	10^{-8} eV/Å	21×21×1	1×1×1
Supplementary Figure 1d	10^{-8} eV/Å	21×21×1	2×2×1

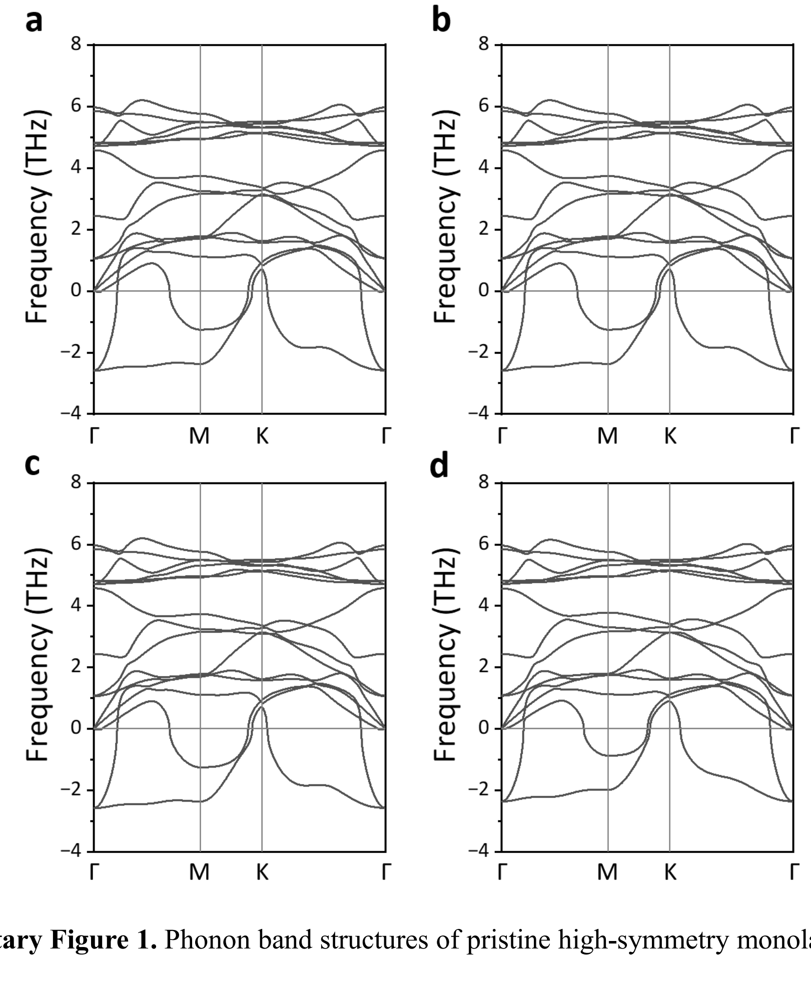

Supplementary Figure 1. Phonon band structures of pristine high-symmetry monolayer β_c-In_2Se_3 generated by different computational parameters for convergence tests and the details for calculations in (**a-d**) are listed in **Supplementary Table 1.** The phonon band dispersion under the four sets of calculation parameters exhibits negligible differences.

Supplementary Note 2. Partial density of states of pristine high-symmetry (β$_c$) and $\sqrt{13}\times\sqrt{13}$ chiral Star-of-David (c-SoD) CDW phases of 2D In_2Se_3.

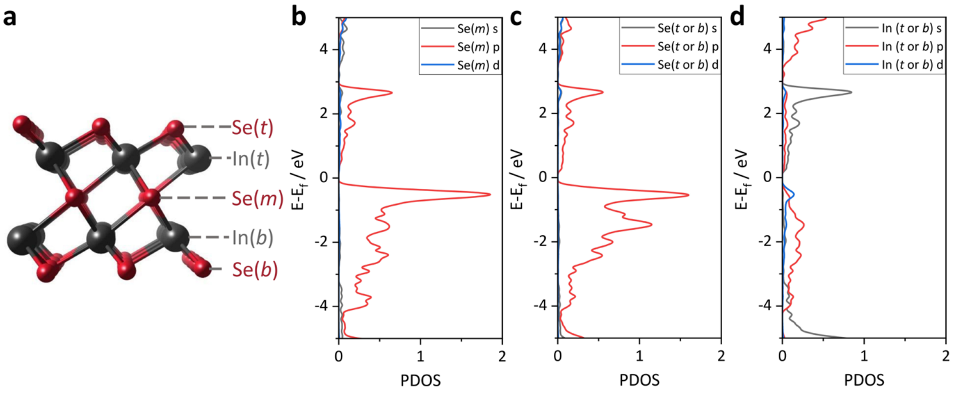

Supplementary Figure 2. (a) Atomic structure of monolayer pristine high-symmetry (β$_c$) In_2Se_3. **(b-d)** Partial density of states (PDOS) of three kinds of atoms. Due to the centrosymmetric of β$_c$-In_2Se_3, the two layers of In [In(*t*) and In(*b*)] atoms are equivalent, and the outermost two layers of Se [Se(*t*) and Se(*b*)] atoms are equivalent.

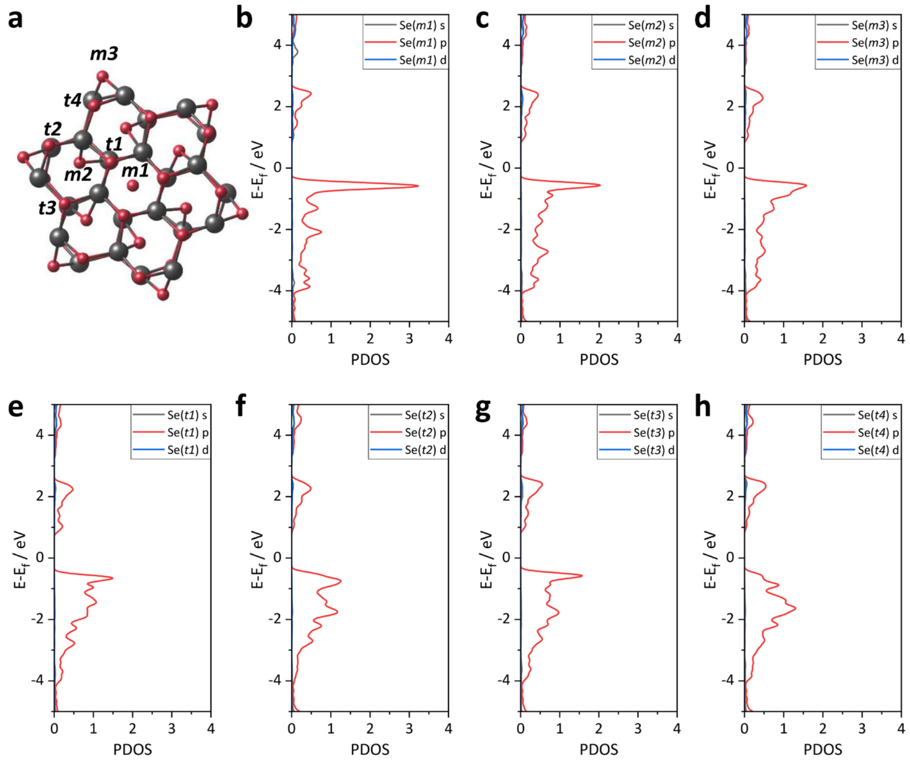

Supplementary Figure 3. (a) Top view of $\sqrt{13}\times\sqrt{13}$ c-SoD CDW phase of monolayer β-In_2Se_3. **(b-h)** PDOS of three kinds of middle layer Se [Se(*m1-3*)] atoms and four kinds of top layer Se [Se(*t1-4*)] atoms.

Supplementary Note 3. Molecular dynamics simulation of monolayer c-SoD In_2Se_3 at 200 K.

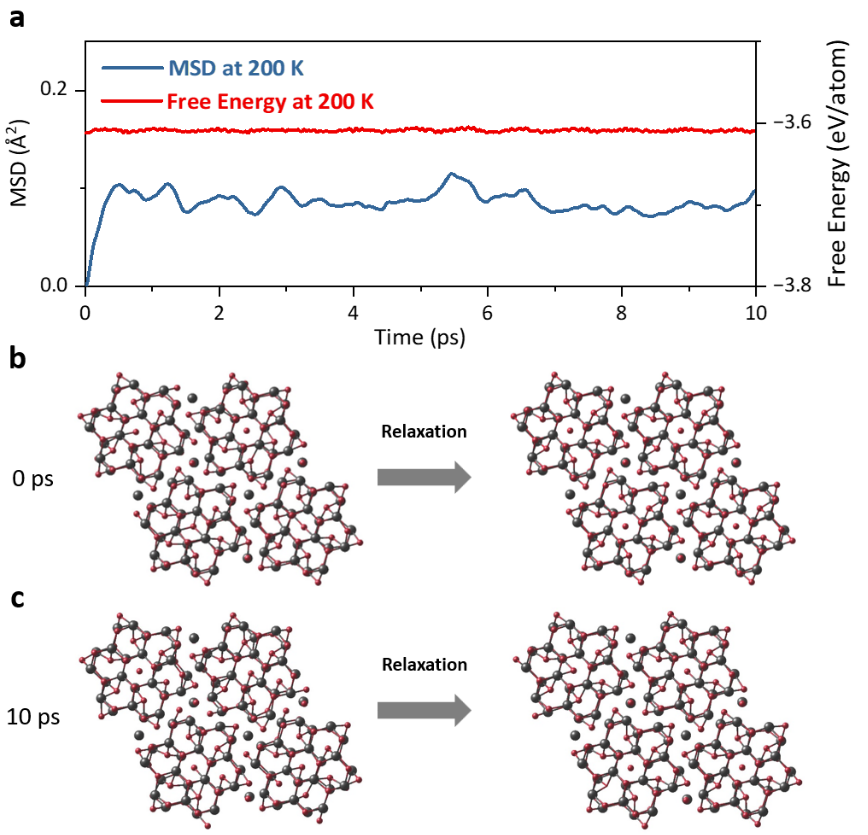

Supplementary Figure 4. Stability characterization of the c-SoD CDW of 2D In_2Se_3 by molecular dynamics (MD) simulations at 200 K. (a) Time evolution of the mean square displacement (MSD) and free energy during the 10-ps MD simulation. Top views of the **(b)** initial and **(c)** final structures, and corresponding relaxed structures of the 10-ps MD simulation.

The temperature of the system was gradually increased from 0 K to 200 K and finally stabilized at 200 K for 10 ps. The time evolution of the mean square displacement (MSD) and free energy of the c-SoD phase are shown in Supplementary Fig. 4a. There are no significant fluctuations in these two curves, indicating that no atomic long-range diffusion or structural phase transition occurred during the 10-ps simulation. Supplementary Figs. 4b and c show the initial and final transient atomic structures and the corresponding relaxed structures of the MD simulation. The c-SoD pattern can be preserved in these four structures, indicating that the c-SoD CDW of 2D β-In_2Se_3 can be stable at the temperature of 200 K.

Supplementary Note 4. Eigen motion analysis of representative phonon modes with imaginary frequencies for 2D β_c-In_2Se_3.

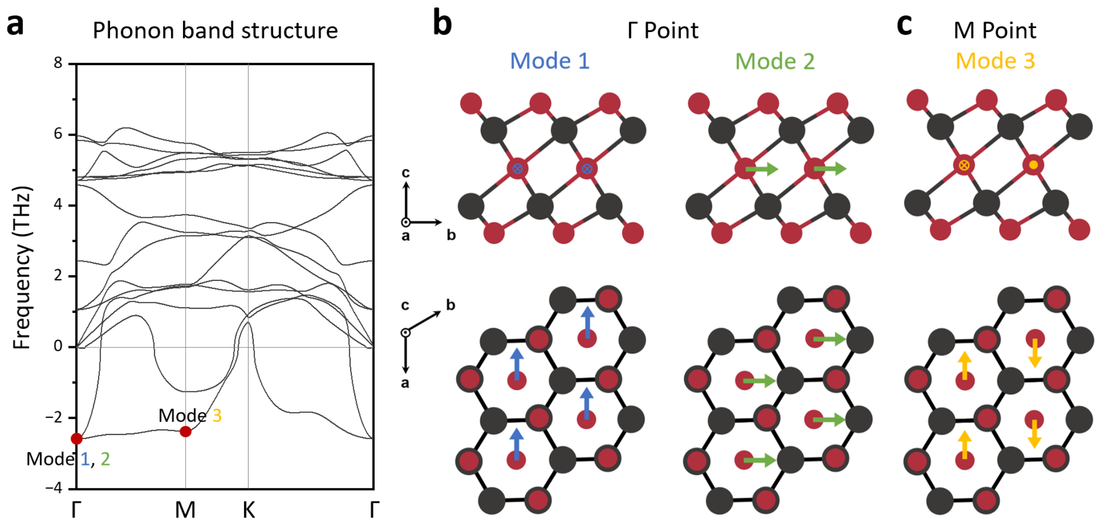

Supplementary Figure 5. Eigen motion analysis of representative phonon modes with imaginary frequencies. (a) Phonon band structure of monolayer β_c-In_2Se_3. **(b)** The eigenmode illustrations of the two degenerate modes (Mode 1 and Mode 2) at the Γ point, marked by a red dot in **(a)**. **(c)** The eigenmode illustration of the Mode 3 at M point, marked by red dot in **(a)**. In **(b)** and **(c)**, the upper row displays the side view, while the lower row shows the corresponding top view.

Supplementary Note 5. Antichiral SoD CDW phase of 2D β-In_2Se_3.

Similar to the ferroelectric phase, the chiral SoD (c-SoD) phase of 2D In_2Se_3 also exhibits directional properties due to the left-handed (LH) rotation and right-handed (RH) rotation modes of the middle layer Se atoms. Therefore, it is possible to construct supercells with different stripe widths that simultaneously contain both LH and RH c-SoD structures, namely, antichiral SoD (ac-SoD_n, where n represents stripe width). The geometrically optimized atomic structures are shown in Supplementary Fig. 6, all these configurations can be sustained, indicating their potential stability. The energy comparison of these three antichiral together with the chiral phases is given in Supplementary Table 2.

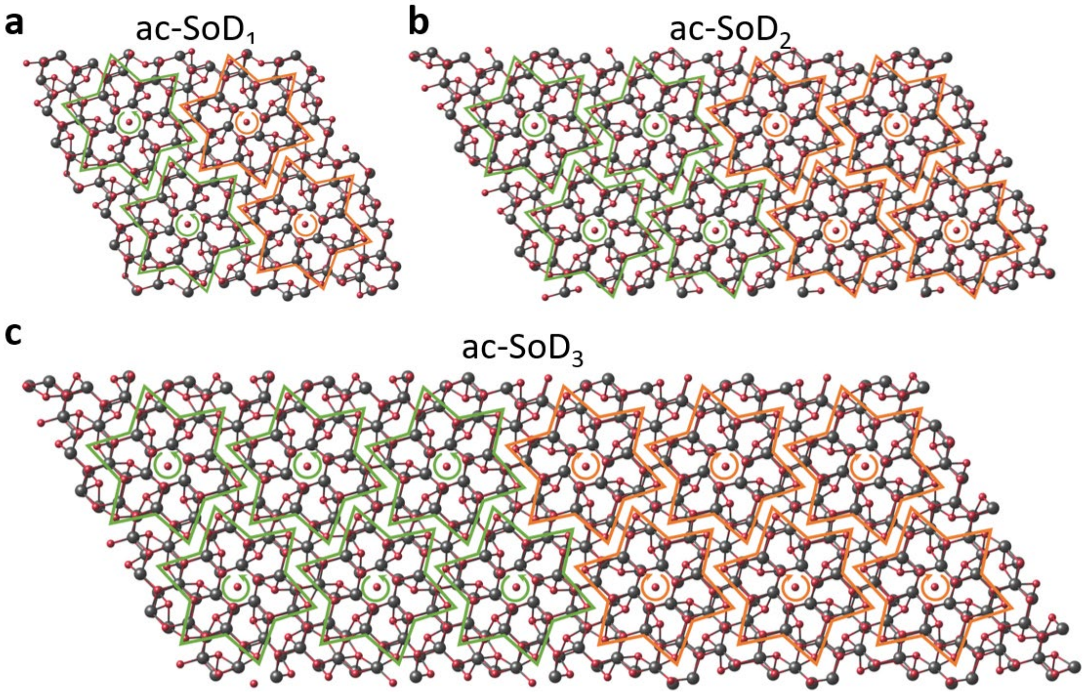

Supplementary Figure 6. Relaxed atomic structures of ac-SoD phases with different stripe widths, namely ac-SoD_n (n=1, 2, 3), **(a)** ac-SoD_1, **(b)** ac-SoD_2, and **(c)** ac-SoD_3 phases.

Supplementary Table 2. Total energies (per atom) of the c-SoD and ac-SoD_n phases with different stripe widths.

Phase	**c-SoD**	**ac-SoD_1**	**ac-SoD_2**	**ac-SoD_3**
Total energy (eV/atom)	-3.687	-3.688	-3.688	-3.688

Supplementary Note 6. The relaxed atomic structures of different CDW orders of 2D β-In_2Se_3.

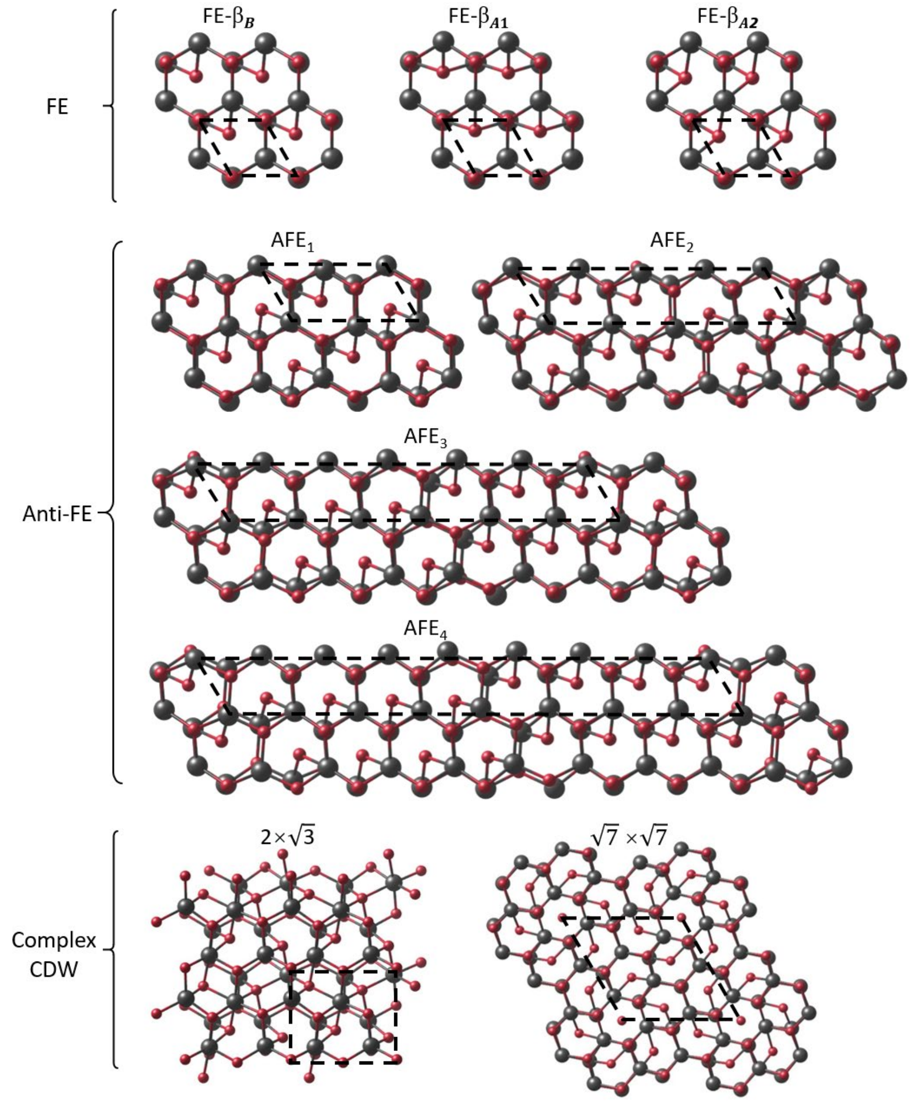

Supplementary Figure 7. The relaxed atomic structure of the ferroelectric (FE), anti-ferroelectric (AFE), and complex CDW phases of 2D β-In_2Se_3.

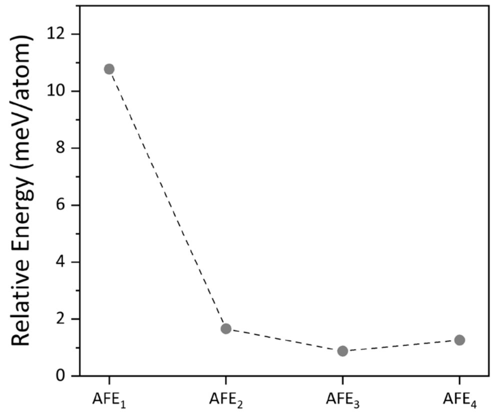

Supplementary Figure 8. The relative energy of AFE_n phases with different stripe widths.

Supplementary Note 7. Density functional theory band structures of 2D c-SoD In_2Se_3 using different pseudopotentials.

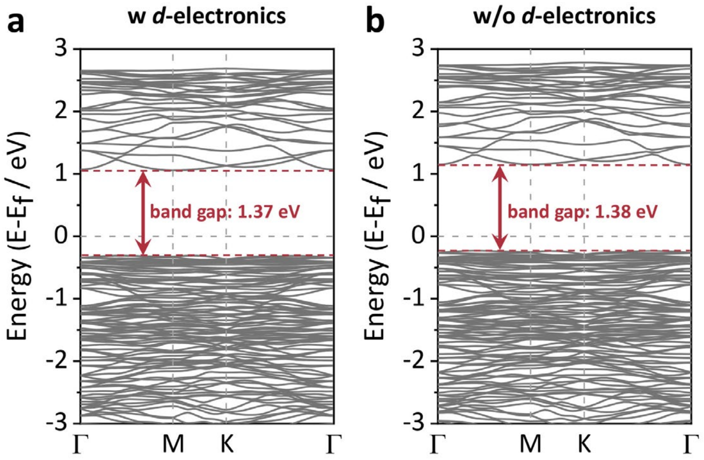

Supplementary Figure 9. PBE band structures using pseudopotentials **(a)** with and **(b)** without *4d*-orbitals of In atom. The displaying two band structures are almost the same.

Supplementary Note 8. Charge density differences of the β_c and c-SoD CDW phases of 2D In_2Se_3.

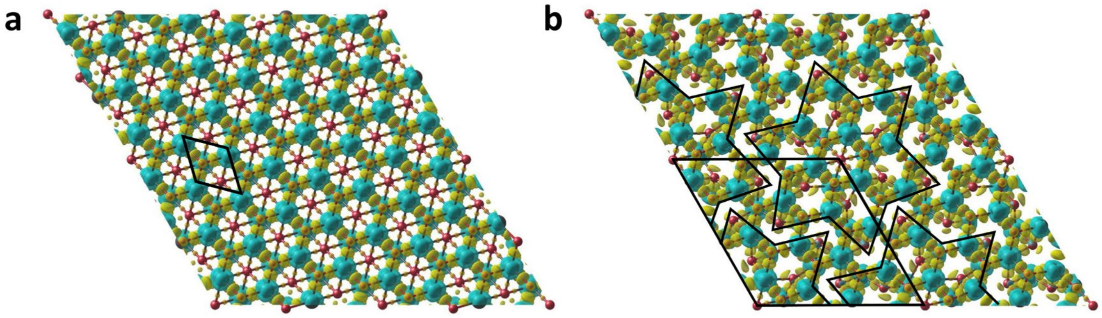

Supplementary Figure 10. Charge density differences (CDD) of the **(a)** pristine high-symmetry β_c phase and the **(b)** c-SoD CDW phase of 2D In_2Se_3. The yellow isosurface indicates charge accumulation, while the blue isosurface indicates charge reduction. The values of the isosurface are 0.0065 e/a_0^3, a_0 is the Bohr radius.

Supplementary Note 9. PBE band structures of different CDW phases of 2D β-In_2Se_3.

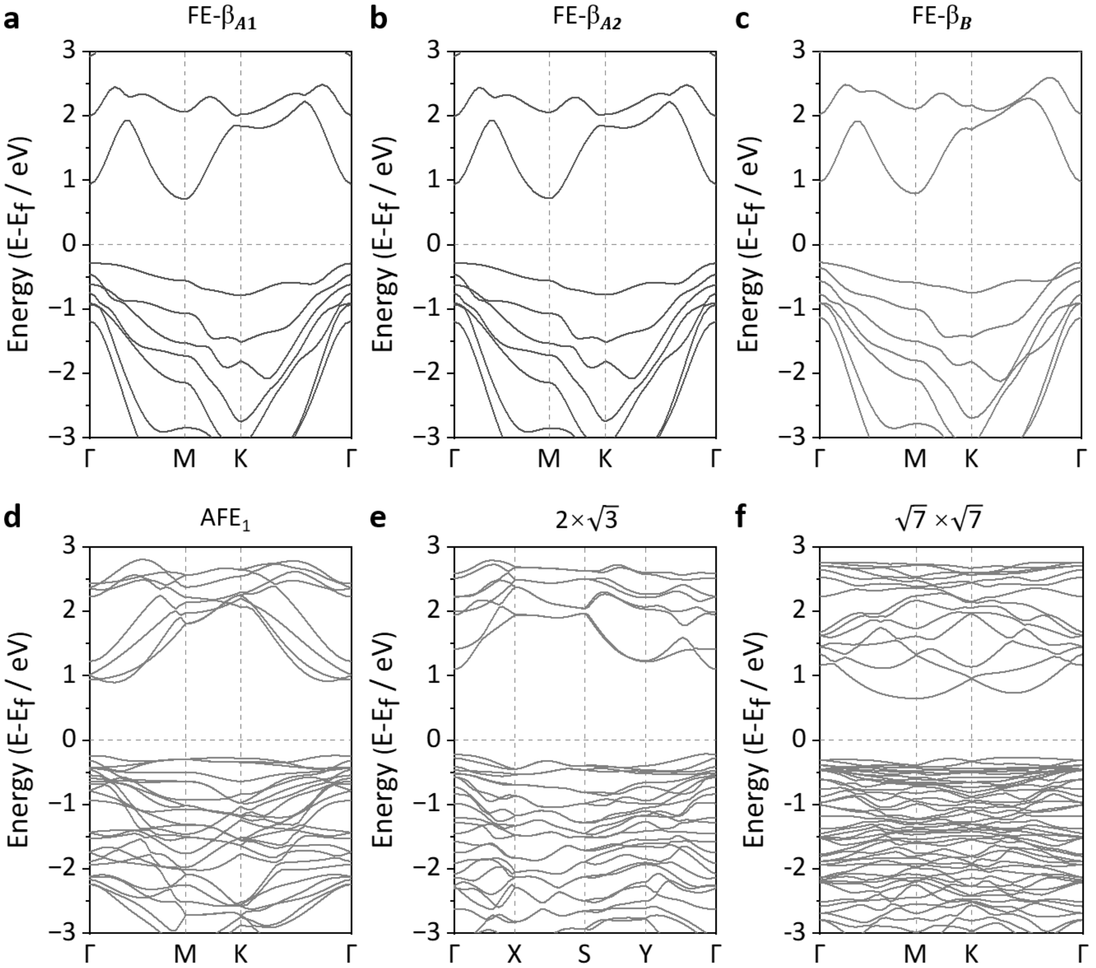

Supplementary Figure 11. PBE band structures of different CDW phases of monolayer β-In_2Se_3. **(a)** FE-β$_{A1}$, **(b)** FE-β$_{A2}$, **(c)** FE-β$_{B}$, **(d)** AFE$_1$, **(e)** $2\times\sqrt{3}$, **(f)** $\sqrt{7}\times\sqrt{7}$ phases.

Supplementary Note 10. Structures of c-SoD CDW phase in nine 2D III_2–VI_3 materials.

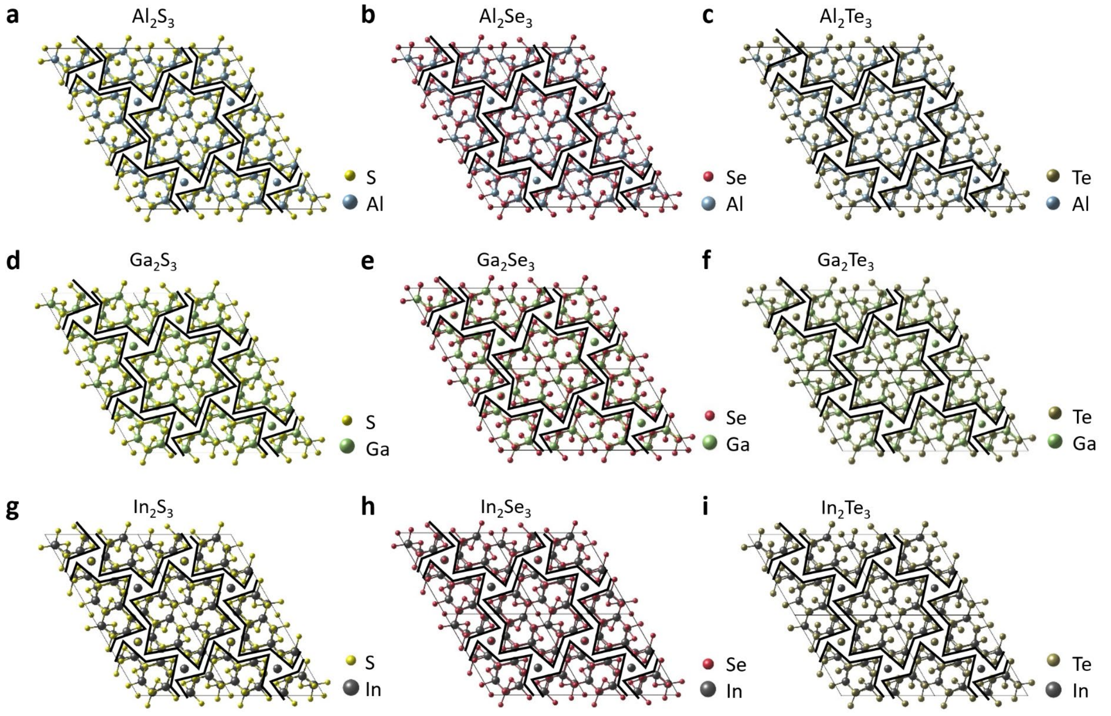

Supplementary Figure 12. Atomic structures of nine $\sqrt{13}\times\sqrt{13}$ c-SoD CDW reconstructed 2D III_2–VI_3 materials. **(a)** Al_2S_3, **(b)** Al_2Se_3, **(c)** Al_2Te_3, **(d)** Ga_2S_3, **(e)** Ga_2Se_3, **(f)** Ga_2Te_3, **(g)** In_2S_3, **(h)** In_2Se_3, and **(i)** In_2Te_3.

Supplementary Note 11. PBE and HSE06 band structures of β_c and c-SoD phases of 2D III_2–VI_3.

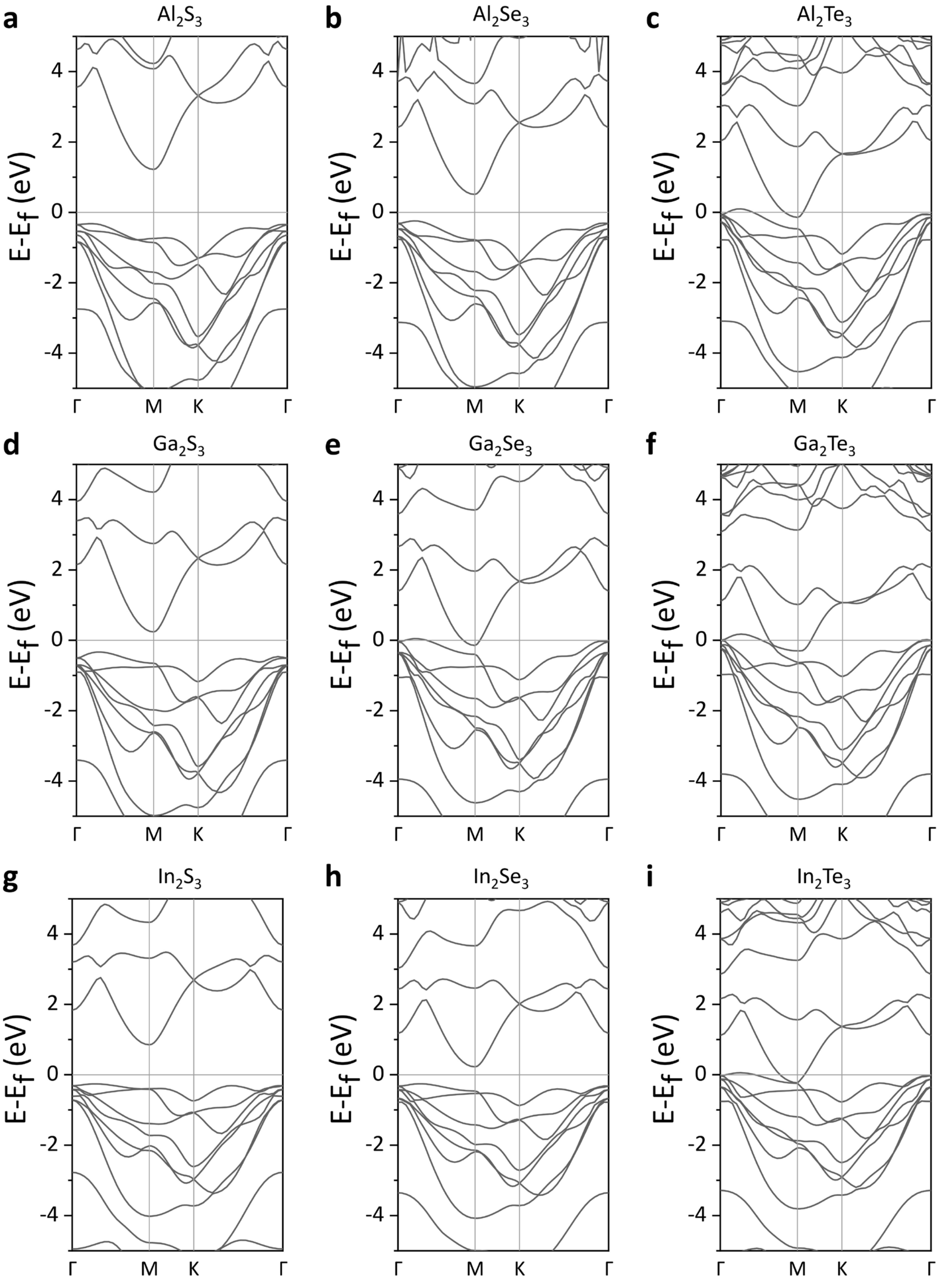

Supplementary Figure 13. PBE band structures of nine pristine 2D β_c-III_2–VI_3. **(a)** Al_2S_3, **(b)** Al_2Se_3, **(c)** Al_2Te_3, **(d)** Ga_2S_3, **(e)** Ga_2Se_3, **(f)** Ga_2Te_3, **(g)** In_2S_3, **(h)** In_2Se_3, and **(i)** In_2Te_3.

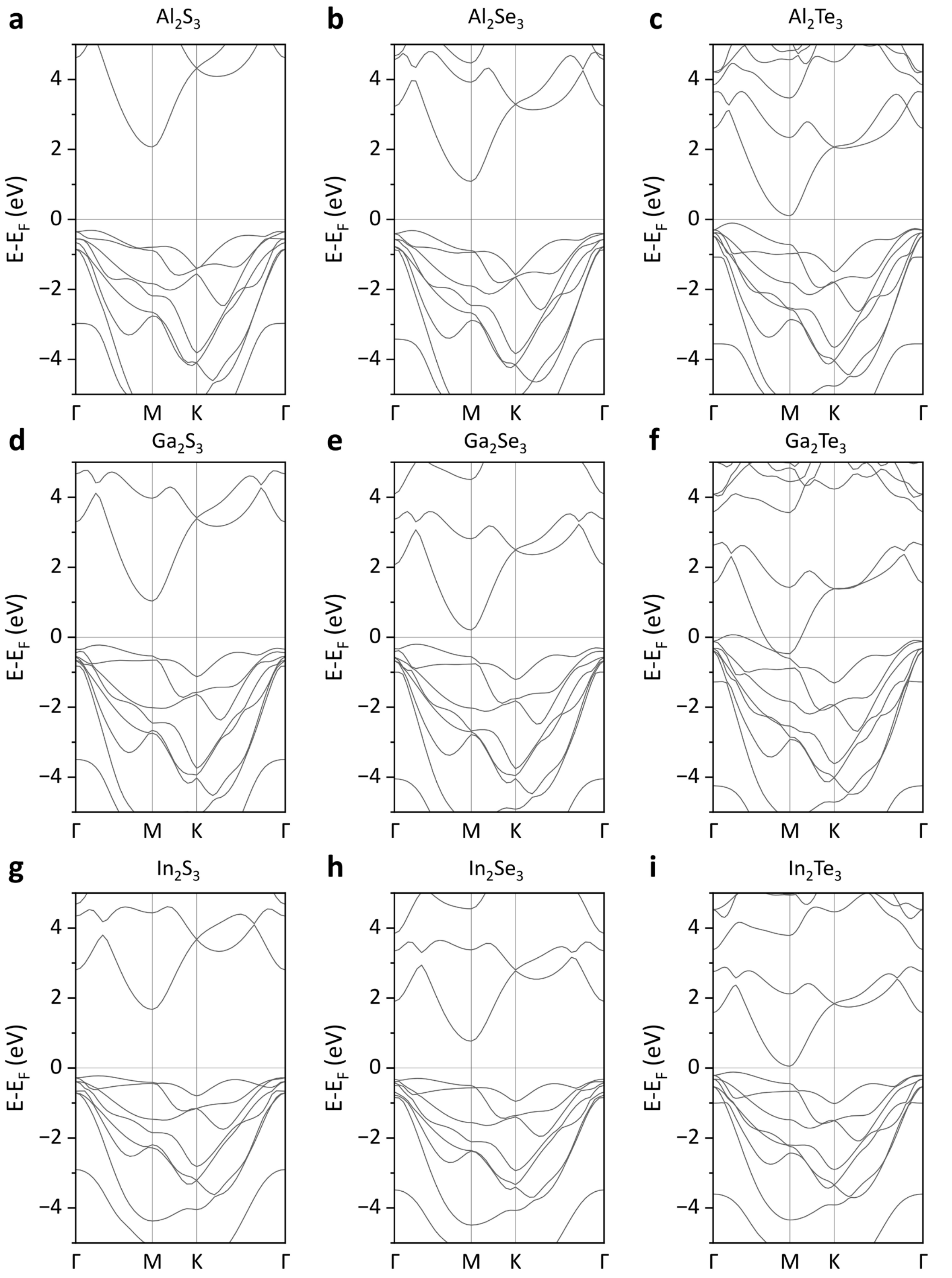

Supplementary Figure 14. HSE06 band structures of nine pristine 2D β_c-III_2–VI_3. **(a)** Al_2S_3, **(b)** Al_2Se_3, **(c)** Al_2Te_3, **(d)** Ga_2S_3, **(e)** Ga_2Se_3, **(f)** Ga_2Te_3, **(g)** In_2S_3, **(h)** In_2Se_3, and **(i)** In_2Te_3.

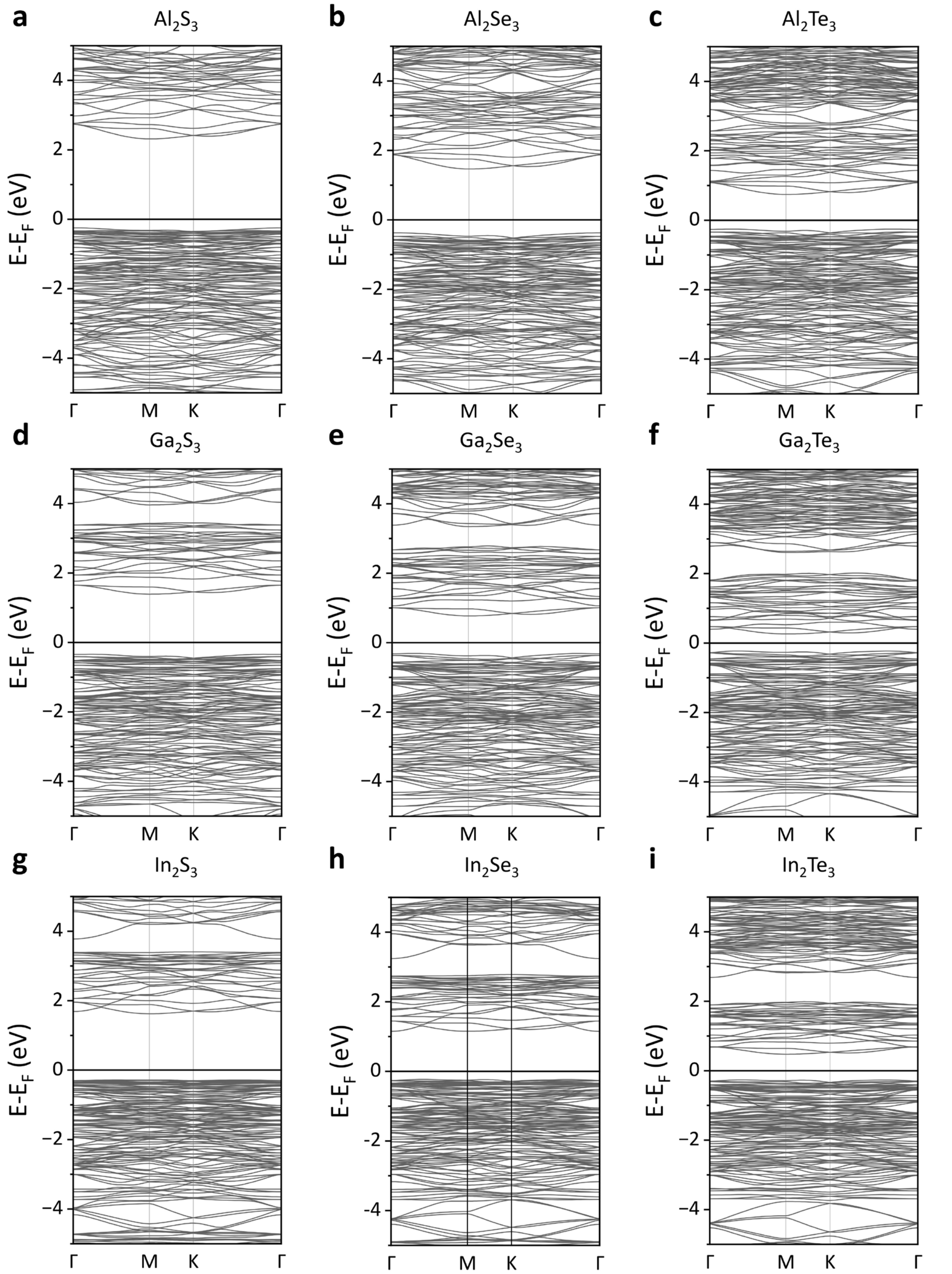

Supplementary Figure 15. PBE band structures of the c-SoD CDW phase of 2D β-III_2–VI_3. **(a)** Al_2S_3, **(b)** Al_2Se_3, **(c)** Al_2Te_3, **(d)** Ga_2S_3, **(e)** Ga_2Se_3, **(f)** Ga_2Te_3, **(g)** In_2S_3, **(h)** In_2Se_3, and **(i)** In_2Te_3.

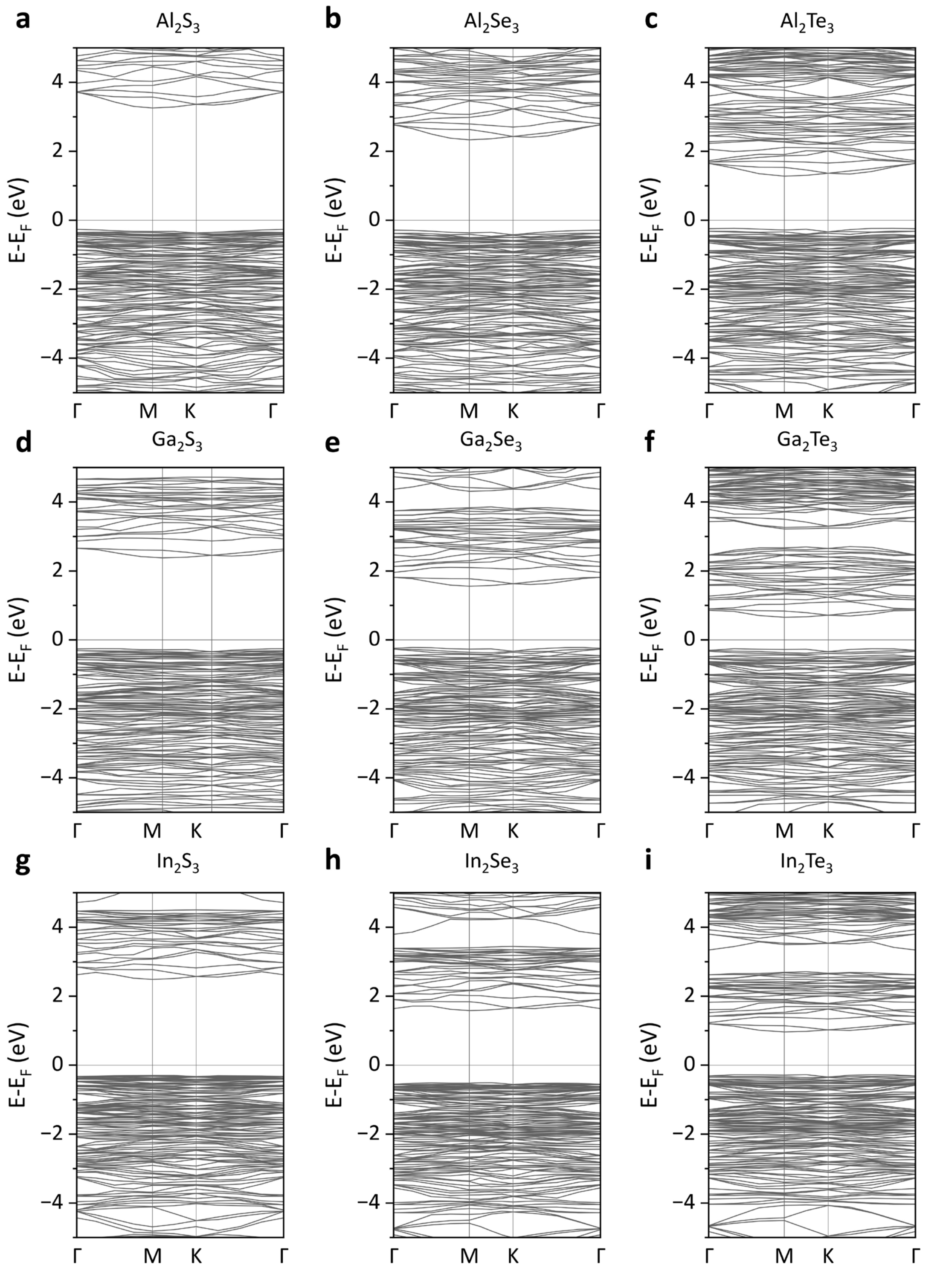

Supplementary Figure 16. HSE06 band structures of the c-SoD CDW phase of 2D β-III_2–VI_3. **(a)** Al_2S_3, **(b)** Al_2Se_3, **(c)** Al_2Te_3, **(d)** Ga_2S_3, **(e)** Ga_2Se_3, **(f)** Ga_2Te_3, **(g)** In_2S_3, **(h)** In_2Se_3, and **(i)** In_2Te_3.

Supplementary References

1. Kresse, G., Furthmüller, J. Efficient iterative schemes for ab initio total-energy calculations using a plane-wave basis set. *Phys. Rev. B* **54**, 11169-11186 (1996).

2. Kresse, G., Joubert, D. From ultrasoft pseudopotentials to the projector augmented-wave method. *Phys. Rev. B* **59**, 1758-1775 (1999).

3. Kresse, G., Furthmüller, J. Efficiency of ab-initio total energy calculations for metals and semiconductors using a plane-wave basis set. *Comput. Mater. Sci.* **6**, 15-50 (1996).

4. Heyd, J., Scuseria, G. E., Ernzerhof, M. Hybrid functionals based on a screened Coulomb potential. *J. Chem. Phys.* **118**, 8207-8215 (2003).

5. Wang, V., Xu, N., Liu, J.-C., Tang, G., Geng, W.-T. VASPKIT: A user-friendly interface facilitating high-throughput computing and analysis using VASP code. *Comput. Phys. Commun.* **267**, 108033 (2021).

6. Tersoff, J., Hamann, D. R. Theory of the scanning tunneling microscope. *Phys. Rev. B* **31**, 805-813 (1985).

7. Nosé, S. A unified formulation of the constant temperature molecular dynamics methods. *J. Chem. Phys.* **81**, 511-519 (1984).

8. Togo, A., Tanaka, I. First principles phonon calculations in materials science. *Scr. Mater.* **108**, 1-5 (2015).
